# Avoidance as a strategy of (not) coping: qualitative interviews with carers of Huntington's Disease patients

**DOI:** 10.1186/1471-2296-6-38

**Published:** 2005-09-14

**Authors:** Alison Lowit, Edwin R van Teijlingen

**Affiliations:** 1 Department of Mental Health, University of Aberdeen, IMS Building, Medical School, Aberdeen, AB25 2ZD, UK; 2 Department of Public Health & Dugald Baird Centre for Research on Women's Health, University of Aberdeen, Medical School, Aberdeen, AB25 2ZD, UK

## Abstract

**Background:**

Since Huntington's Disease (HD) is a familial disease with an average onset in the mid-thirties, one might expect that spousal carers are concerned with providing care for off-spring who may turn out to be affected.

**Methods:**

This study involved ten face-to-face interviews with carers of spouses affected by HD in Northeast Scotland. Carers were recruited through two channels: a genetic clinic and the Scottish Huntington's Association (SHA). Interviews were conducted in carers' own homes. A thematic analysis of the transcripts was conducted.

**Results:**

Although carers did worry about their children, they did not envisage being involved in their care. Many avoided talking about the disease, both within and outwith their family; this may have greatly reduced the level of support provided by family members. Conversely, avoidance was often accompanied by symptom-spotting. For example, several people had given up driving, before they were incapable of doing so. The explanation appears to be that they avoided getting into situations in which HD may express itself.

Support meetings seem to be valued amongst patients with other serious diseases and their carers, however, although all participants had had contact with the SHA, only one regularly attended meetings. It was felt that seeing others with HD provided a constant reminder of the possible effect of HD on the wider family, which seemed to outweigh the benefit of attending. Overall, the analysis highlighted 'avoidance' as a key theme.

**Conclusion:**

Many denied symptoms of HD in their spouses, pre-diagnosis. All had pretended at some point that it was not happening, through ignoring early signs and 'obvious' symptoms. Some partners had refused to go to the doctor until it was no longer possible to deny symptoms. Formal health and social care seemed to play a very small role compared to informal care arrangements.

## Background

Huntington's Disease (HD) is a terminal inherited progressive neurodegenerative disorder of the central nervous system, characterised by a variety of symptoms that affect the patient's physical and mental health. Folstein [1] describes the disorder as having a "triad of clinical features", because it has a range of motor, cognitive and psychiatric symptoms. Symptoms include personality change, movement disorder, cognitive impairment and mental health problems [1]. The average duration of the disease is 16 years [2], but it can vary greatly; a range of 4 – 38 years was documented in Folstein's study. [1] Symptoms can start at any age, but typically occur between the ages of 35–40, often after the affected individual has reproduced and possibly transmitted the faulty gene to his/her offspring. Inheritance is autosomal dominant and each child of an affected person has a 50% risk of inheriting the condition and developing HD. [2]

The gene causing HD (IT15) was isolated in 1993. Prior to this, the only possible method for presymptomatic HD testing was by linked DNA markers. This was a complex procedure that would not always give a conclusive result, and needed blood samples from multiple family members. The isolation of the single specific mutation meant testing became possible, with a reliability of >99%, without the need for co-operation of relatives. This has been of profound importance to HD family members, allowing a simple and accurate molecular test to determine their HD status. [2]

There are no treatments that can cure, delay onset or slow the course of HD; from onset onwards progressive degeneration occurs and the sufferer requires increasing levels of care and maintenance [2], but provision of a "full range of supportive medical nursing and social care" can help improve the patient's quality of life [28]. The complex characteristics and symptoms of HD mean caring for an affected individual can be particularly stressful and problematic. HD usually affects an individual at a time when they have many responsibilities to their family; this means the partner who acts as carer is often placed in the position of total responsibility, taking on the roles and responsibilities of their partner as well as their own. [3] HD places a great burden on the primary carer. Due to the hereditary nature of the disease, HD does not disappear with the death of the affected individual, but can repeat itself in successive generations. Consequently a carer may care for more than one generation of sufferers. [4]

Due to the rarity of HD many GPs (General Practitioners) and health workers do not have any real understanding of the disease, or the needs of patients and their carers. [5] An inadequacy of facilities for this population is very common, and management of the illness is very difficult. The complexity of the symptoms means that the HD patient is "unable to fit into the system". [6] Because HD patients' present symptoms that cross many different health and social care disciplines, there are often logistical and co-ordination problems associated with care provision. To be useful care has to be delivered timely, flexibly and in a co-ordinated fashion, to meet the patient's immediate requirements. All too often this does not happen, and the carer ends up being the central person in the day-to-day management of the patient.

This qualitative study was undertaken to gain in-depth understanding of the carer role in HD in the Northeast of Scotland; exploring the issues carers perceived were important when faced with the prospect of caring for one or more family members affected by HD. The study explored carers' perspective on their care-giving role and how that role had changed their lives. The themes explored were: motivations for caring and coping mechanisms adopted, plans for caring for future generations of HD relatives, utilisation of support services and facilitating factors and barriers to providing effective care.

## Methods

The primary goal of qualitative research is to develop concepts, which help to understand social phenomena, emphasising the views, experiences and meanings of people experiencing the phenomena [7]. In order to answer the research question it was important to explore people's attitudes, views and experiences, therefore face-to-face interviews were selected as the most appropriate qualitative method. With the semi-structured interview there are no assumptions made or theories to prove; the method begins with an area of study and allows relevant data to emerge during the research process. [8] This type of interview aims to be like a guided conversation with a purpose. [9,10]

Ethical approval was applied for and was granted by the regional Medical Research Ethics Committee. Permission to approach clients was sought from the regional HD genetic clinic and the Scottish Huntington's Association (SHA). Due to the time scale of the study a convenience sample of ten carers was recruited from the Dundee, Angus and Grampian areas (the Northeast of Scotland).

Criteria for participant inclusion were that they (a) spoke English; (b) provided hands-on care for a family member who had been diagnosed with HD; and (c) were not participating in any other on-going research. A letter of invitation to the study, explaining the background to the research and the carer's potential role in the study was given to potential participants by a SHA adviser or the clinician at the regional HD genetics clinic, along with an acceptance slip and stamped, addressed envelope. Replies were sent to the first author at the university, who then arranged the time and place for the interview. Ten potential participants were approached and all agreed to be interviewed.

All the interviews were conducted in the carers' own home to make them feel more at ease. As many feel it is important to create a relaxed atmosphere in a comfortable setting to promote the development of trust. [9] Immediately before the interview the researcher explained the purpose of the study, the method of recording and analysing the data, and answered any questions the carers had, before obtaining their consent. When the study was completed participants were sent a summary of the findings.

Each interview started by asking the participants who they cared for, and what they did as a carer. This allowed participants to tell their own story and formed a starting point for the interview to develop. Themes were explored using open-ended questions. Questions were changed, dropped or expanded upon depending on each individual's experiences. The interviews were tape-recorded, [10] and transcribed verbatim within 24 hours, i.e. as soon as possible as the interviewer is more likely to recall exact details. [9]

The analysis consisted of reading and re-reading the transcripts to identify themes, patterns, salient points, common threads and trends, and to search for deviations and exceptions to these trends. [11,12] After the main themes were identified, each separate theme was explored individually to produce the final findings.

Several of the themes identified and explored in these interviews are beyond the scope of this paper (see Table 2). This paper focuses particularly on the theme of 'avoidance', and the part it plays in the coping strategies adopted by HD carers. Themes are presented by quotes from the interviews, which have been labelled with only an interview number to avoid possible identification of individual interviewees.

**Table 1:** Background data on carers/interviewees and patients

**Carer**	**Patient**	**Relationship to Patient**	Children*
Male, (60–69)	Female, (60–69)	Husband	3
Male, (50–59)	Female, (50–59)	Husband	2-carer; 3-patient**
Male, (80–89)	Female, (70–79)	Husband	0
Female, (40–49)	Male, (50–59)	Wife	1 each**
Female, (50–59)	Male, (50–59)	Wife	3
Female, (40–49)	Male, (40–49)	Wife	3
Female, (60–69)	Male, (60–69)	Wife	4
Female, (50–59)	Male, (50–59)	Wife	2
Male, (50–59)	Female, (40–49)	Husband	2
Female, (40–49)	Male, (40–49)	Wife	0

* Number of children per couple have been slightly changed to maintain anonymity.

** Both have children from previous marriages only

## Results

The ten carers were interviewed, were between 40 to 90 years old; six were female and four male. They were all spouses of the affected person. All but one carer cared for their spouse at home. The one carer, whose spouse was in a nursing home, had up until eight months ago, cared for her husband at home. The duration of the relationship between carers and spouses ranged from nine to 42 years. All stages of HD were represented in the sample. Table 1 summarises the sample.

The families in the sample had 24 children in total. Thirteen children were still at 50 percent risk of having the HD gene, a further eight had been tested for the HD gene; five are negative and three are positive for the faulty gene.

Only two carers knew about HD in their spouse's family, and the implications of the disease, before they were married. The partners in one couple had both been married before and knew the spouse had HD prior to the marriage. The other couple married not knowing the spouse's HD status, but decided not to have children.

One carer had been told that there was HD in the spouse's family, but had been told that there were no implications for them as a couple. One carer suspected HD in the spouse's family, although the family themselves were unaware of it. Six carers did not know about the existence of HD in the spouse's family prior to marriage. Three found out before their spouse's diagnosis and three found out later.

**Table 2:** Additional themes identified in interviews

**Themes**	**Examples**
Motivation for being a carer	• Marriage vows • Memories of past life together
Changing roles and relationships	• Demise of reciprocal loving relationship • Loss of independence
Coping mechanisms	• Living environment • Slow course of HD
Employment & financial changes	• Loss of employment • Lower income
Experiences of social/ health care services	• HD management • Respite care
Scottish Huntington's Association	• Practical advise • Emotional support
Living with HD	• Impact of the disease • Personal development of carers

### Impact of HD

HD has implications that extend far beyond the patient. One carer summarised the impact of HD on her spouse's family. Speaking about her father-in-law she said:

"He knows what's happening because his wife died with it. It's hard for him because they are his children. Three of his family have it and some of his grandchildren. It's not easy. (Husband's name removed) older sister has it and her son has it, though he doesn't have symptoms yet. His other sister has got it, but we don't know if any of her children have got it, they decided not to be tested. His younger brother we don't know about yet, because he's still quite young – in his thirties, and he hasn't been tested. He has children too. It's hard. It takes over everything, because quite a lot of his family are going to have to go through this, some are already." (Interview 4)

The initial diagnosis of HD was very difficult for those carers who had some knowledge of the disease. For some the shock lasted a long time:

"When we first found out he had it, it was hard. I was traumatised for quite a while afterwards." (Interview 6)

One carer, who had not been aware of HD, believed her ignorance had not made much difference to the way she handled her situation. She said:

"I don't know what I would have done – you may think I would have been better prepared – I might have been financially because we certainly were not – it was a nightmare, but nothing can prepare you for this. I don't believe there is anything that can prepare you for the horror of HD. I just wouldn't wish it on anyone. I worry mostly because of the children." (Interview 5)

### Avoidance

Avoidance was a strong theme that was manifest in family members involved with HD. Avoidance often took the form of refusal to discuss HD within the family. One carer whose spouse had been diagnosed said:

"Well you know, he wouldn't talk about it at all. It was difficult to find anyone to talk to within his family. I didn't know about it (HD) and they weren't going to tell me anything about it." (Interview 5)

The five carers who knew that their spouse was at risk before their diagnosis also said that they avoided talking about or thinking about HD:

"We just got on with our lives without thinking about it. We never discussed it. What was the point? He could just as easily not have had it. A 50 percent risk is a 50 percent risk!" (Interview 10)

The main motivation behind refusal to discuss the issue in couples where risk is known seems to be a form of denial. Carers wanted to believe that their spouse was not affected by HD, and avoiding dealing with the risk factor reinforced this.

"I put it in a little box in the back of my mind because really I was hoping she wouldn't have it." (Interview 1)

For some, avoidance can be so strong that denial of obvious symptoms makes the diagnosis even more distressing, as the following interview highlights:

"After seeing her mother, well I had spent so long hoping she didn't have it and kind of ignoring her symptoms, that when I was told it kind of knocked me for six." (Interview 9)

After initial diagnosis, this inability to discuss HD continued for all the carers. For some, discussion seemed pointless in the face of inevitability, which of course, is not exactly the same as avoidance:

"I just think you have to keep going as normal as possible for as long as possible. Why discuss it – what's the point?" (Interview 8)

There was also a lot of secrecy surrounding HD within families. The secrecy was also present within the spouse's own family. This secrecy may have contributed to the inability to speak about the disease. One carer who believed that she had been kept in the dark about the implications of the disease was very angry and resentful:

"I was so angry – I felt angry with him and his dad. I suppose I wanted to protect my children. I wanted it to be all right for them. I was angry. He said he didn't know the implications of the disease, but I'm sure he and his dad knew more than they told me. Looking back I am sure of it." (Interview 5)

Another carer knew an hereditary disease existed in her partner's family, but had not been given any details about the disease. She suggested that if she had known, she might not have married her partner. Avoidance manifests itself at different levels – HD was not hidden from her, but no details about the disease were provided and this carer did not seek information for herself:

"I never met his dad, his mum never spoke about it, neither did he........ I knew when we married that this disease existed in his family, but I can honestly say I never thought about it. As I say, I had never met his dad, maybe if I had it would have been different." (Interview 10)

Although family secrecy was the cause of distress and anger for some carers, avoiding confronting the existence of the disease and discussing its implications sometimes led to a repetition of secrecy in the next generation:

"Well we never spoke about HD, never spoke about that, the kids never knew about it." (Interview 8)

Conversely, although all the carers talked about an unwillingness to talk about HD, several mentioned that they did look for the first signs of HD symptoms:

"I started noticing slight things about six years ago. I often wonder though, if I would have noticed them if I wasn't aware of the Huntington's." (Interview 4)

Symptom spotting and avoidance are conflicting behaviours:

"When I spotted the first signs I just kind of hoped that it wasn't it. I suppose I knew it was, but still I didn't think about it at the time." (Interview 9)

Recognising the symptoms, may have affected the way the spouses behaved. Some patients avoided initial diagnosis. One carer stated:

"It was a long time before she would go and see a doctor. A very long time, I had to persuade her to go because she knew herself what was coming and just didn't want to know. I'm sure it was that." (Interview 1)

Some spouses avoided situations where their failing abilities might be exposed. For example, four of the carers said that their spouses had given up driving, long before they had been diagnosed:

"He felt he was having problems driving and I was getting a bit worried about the driving situation...but he just gave up driving – I did all the driving." (Interview 10)

None of the couples indicated that they prepared for the long-term future or planned for the care of future generations of HD sufferers. Owing to the untreatable nature of HD, and its inevitable progression, avoidance and denial may have been part of coping mechanisms. Avoidance also influenced carers' ability to plan ahead. Their strategy was quite simple, they just dealt with problems as they arose:

" You can't really plan for it – I just take one day at a time. It's so slow you just adapt. I don't make plans." (Interview1)

Although the six couples who had children together all believed that the risk of transmission to their children was the worst part of the illness, they still avoided serious consideration of the long-term implications of HD:

"The implications for my children, that's the hardest thing. I find it so difficult to cope with. In fact I don't cope with it." (Interview 6)

The carers with children at risk were asked if they thought that they might be involved in the care of their own children. All but one carer said that it was a subject they never thought about, and they certainly didn't prepare for it. Typical responses were:

"No – I don't think about that (caring for child), not at all, never. It's not something you can think about." (Interview 8)

And

"I haven't thought about that – no. I hope they haven't got it. That's all I think about it." (Interview 9)

The one carer who had given the matter serious consideration had an affected daughter. However, for personal reasons she was not actively involved in caring for the daughter.

Although all the children knew of their risk, once the initial questions were answered all but one carer said that they never talked about it again:

"They know their dad is ill and it's going to get worse, and they know that they are probably at risk of getting it as well. And to me that's enough for them to be getting on with. We didn't talk about it because, for me, that would just pile it on." (Interview 8)

Avoidance was a strong theme here also:

"I don't really talk about it with them (children). We have spoken about it, but not now. Maybe I just hope they haven't got it." (Interview 9)

### Issues around testing

All the carers with children at risk from HD said that they didn't put any pressure on them to be tested for the HD gene. All thought that this was a decision that their children should make for themselves. Of the children at risk less than half had undertaken the test. First hand knowledge of the symptoms and course of the disease acquired from close proximity to an affected parent, appears to influence the decision not to take the test. One carer, discussing or perhaps justifying why her husband had not been tested, explains:

"He lived through his father's illness from start to finish, so he didn't want to be tested. He said that he didn't want to know, it had been so awful that he wouldn't have been able to cope...knowing what was in store for him." (Interview 10)

There was also some concern that the genetic implications made the relationship between the children and the affected spouse distant. One carer explained:

"It has been difficult for them, seeing their dad change and what that might mean for them. They all find it difficult to be with him...it's not just the change in their father, it's partly that, but it is also that they see what might be in store for themselves." (Interview 7)

Problems arose both when a child had been tested and when it had not. One carer whose daughter had tested positive said:

"My eldest has been tested and is positive, but she doesn't want anyone to know. I wonder how she feels (daughter) knowing she has it. She won't talk about it. I think she has hidden it away." (Interview 6)

### Lack of support for carers

The carers felt they had a close relationship with their children, and that relationship helped them emotionally and greatly enhanced their quality of life. However none of these children were actively involved in the care of their affected parent. One of the two offspring still living at home was an adult, but she did not help with any of the care of the affected parent, or upkeep of the house. This caused some friction within the home:

"We have a daughter who stays here. She doesn't lift a finger – she's 26, nearly 27, but she doesn't help either of us at all. I don't understand that, we argue about it all the time." (Interview 9)

None of the carers got any help from their spouse's parent. Several of these parents visited but none were involved in the day-to-day care. One carer tried to explain why that might be so – she stated:

"She had many problems looking after his father, especially as she had two children to bring up at the time. And really, I don't think she wanted to be involved in his care. She visits frequently, but doesn't help with his actual care." (Interview 10)

Another carer believed that it would be a great help if her spouse's mother could help her for a short time each week. She explained; this was not on offer:

"I think his mother should help me more. If she could take him out for a couple of hours a week that would really help. But I've asked her about that and she just shouted me down. She thinks it is my duty to look after him. She has no intention of giving me help." (Interview 6)

Several of the carers had no contact with their spouse's wider family and those that did said that it was just on a social basis that only happened once or twice a year.

### SHA

This avoidance of contact with other HD families was also evident in the low attendance at support meetings. Although all the carers valued and praised the work of the HD voluntary service, all but one of the carers had stopped attending support meetings. They believed that, rather than helping them, meeting other carers made things worse, because they were presented with a vision of their future:

"Well I don't go. You kind of see what's in store for you, and it's not nice. Knowing – well, seeing what's going to happen. I found it too depressing."(Interview 7)

Regular attendance at SHA meetings did not fit in with the "one day at a time" attitude most carers had adopted:

"I went to one meeting, but they were all so miserable that I felt worse when I came out. I don't really want to know what's in store for me. I'd rather just cope with things as they happen." (Interview 10)

## Discussion

The carers in this study had willingly accepted the responsibility of caring for an HD patient, and seemed to care with dedication and conscientiousness. The carers coped with the problems they encountered in day-to-day life due to their spouse's HD, by accepting the disease and trying to develop strategies to deal with the problems as they arose. The slow, progressive nature of HD facilitated this. This has been documented in studies exploring the caring role in other slow progressive diseases. [13-16] Many studies reporting on caring for family members with long-term progressive illnesses report that carers express a need to meet and share experiences with people facing the same type of situation. [13,17,18] However, the majority of carers in this study did not attend support meetings or wished to meet other HD carers.

The main reason given was that they did not like being presented with a vision of their own future. However, this does not adequately explain the contrast of attitudes between HD carers and carers of other progressive, degenerative diseases. Such carers are often active in attending support group meetings, where they too will be presented with a vision of their future. Given the hereditary nature of HD, the vision being presented may be more than simply the couple's own future, but also an unwelcome reminder of what may be in store for other family members.

Unlike other illnesses, which run a course and then disappear, albeit in death, HD affects whole families for generations. The hereditary nature of HD seems to impact on all aspects of life. Family breakdown frequently occurs, secrecy can be so great that it becomes impossible to discuss the subject within the family, and each member lives in isolation with his/her own feelings about the disease. [19] This may be because the scale of the problem witnessed in the sufferer is magnified by the genetic implications. Several studies found that many HD families adopt denial tactics to cope with the disease. Hiding the existence of HD within the family; not talking about HD, avoiding affected family members and denying one's own or one's spouse's symptoms will in some way avert the impending doom. [3,20] Refusal to allow information to be given to children has also been reported. [21,22] Evidence suggests that this inability to talk about HD is one of the reasons why the information about the disease held within families is so distorted, inaccurate and incomplete. [2,4,22]

Avoidance also affects support within the family. In this study none of the carers were getting any help with the care of their spouse from family members. Children, although close to the unaffected parent, were not involved in caring for their affected parent. The affected spouse's family also had no active involvement, and surprisingly the carer's family were not involved in helping with the affected spouse. Research has suggested that the burden of guilt and anxiety carried by members of HD families diminishes their ability to help and support affected individuals. [2,23]

Living with HD is essentially learning to live with stress, anxiety, fear and loss, which may help to understand the high level of avoidance and secrecy that existed within these families. Secrecy may result from parents' desire to protect children, which in turn may result in fragmentation of the family network as they avoid family members who have the disease, or are aware of the implications of the disease. Several of the carers had no contact with the spouse's wider family.

Once the initial diagnosis or "at risk" status was known and initial enquiries had been answered, HD was not mentioned inside or outside the family. The multiple problems which impinge upon all aspects of daily living, and the long-term prospects, may be too distressing to contemplate, so the full reality of the disease is hidden away and problems tackled only when they arise. However, this conscious avoidance is accompanied by symptom spotting and avoidance behaviour (such as giving up driving long before real symptoms arise), which suggests that although HD is not consciously spoken about, subconsciously it is ever present. This confirms previous reports on the effect of the all-consuming nature of HD on the affected families. [2,20,23,24]

By far the greatest problem for carers with children was the fear of their children developing the disease; all found the task of telling their children of their risk devastating. Once children had been informed of this, HD was not a topic that was openly discussed again. Protecting the children seems the main reason for this – speaking about HD would only make it more upsetting.

Only a small proportion of children had been tested for the faulty gene. This is consistent with the normal trend in HD families. [2,25,26] Before an accurate predictive test was available, continuous anxiety of asymptomatic family members lead to a belief that an accurate predictive test was urgently required and would be welcomed by HD families. However, the anticipated high uptake of the test has not been realised. [2] This may be due to asymptomatic individuals preferring to live with a 50 percent risk of having an incurable, degenerative disease, whose symptoms and course they are all too familiar with, rather than taking the test, which may conclusively indicate the presence of the disease.

Given the 50 percent risk each child and sibling of an affected individual has, the expectation was that carers might have considered being involved in care provision for other affected family members, and in anticipating future care they might have thought about how that care could best be provided. However, all the carers stated that this was not something they considered or prepared for. For them, this was something to deal with in the future, and certainly not a subject that they thought about or prepared for. The one carer who had an affected daughter stated that she would not be taking on the role of full-time carer for her daughter.

The study by Brouwer-Dudok de Wit and colleagues. which assessed the psychological distress of partners of people with late onset hereditary conditions revealed that partners of individuals at risk for HD had significantly higher levels of avoidance than partners of people at risk for hereditary cancers. [20] The ways in which individuals cope with stress has been extensively studied. [27] Avoidance is recognised as part of the coping process when individuals are confronted with threats they cannot deal with directly. [28] An individual judges a situation as traumatic and senses a painful effect such as depression or helplessness, so diverts his/her attention to something less painful. It is seen as a protective mechanism that allows us to proceed with life in greater psychological comfort. [27] Because HD is an incurable genetic disease combining mental and physical deterioration, the future may be too disturbing, and prognosis too hopeless, for carers to contemplate.

Avoidance and denial may be helpful in (temporarily) circumventing a serious problem but there is evidence that denying or suppressing painful thoughts can actually result in higher levels of anxiety and distress. [29] When a disease is serious, progressive and terminal, avoidance and denial may have serious consequences for the long-term well being of the carer. In such cases avoidance and denial may interfere with the maintenance of supportive relationships with partners, relatives and friends, and may preclude any form of long-term preparation. Several of the carers stated that they had no contact with their spouse's family; their children had very little input into the care of the affected parent and generally they felt distanced from any close supportive help with the day-to-day management of the condition. Skirton and Glendinning have argued that families could be supported and assisted in caring for their relatives in the home if appropriate. [30] We feel that avoidance and denial in some carers might prevent them from seeking and/or accepting such external support.

This study suggests that HD carers adopt an operational, or at best tactical approach to care, rather than a strategic one. Their considerations focus mainly on the immediacy of their current situation; they cope with their role by solving problems as they arise rather than planning coping strategies for future events.

Caring for an HD sufferer requires dedication and commitment, and it is a full-time occupation. At the same time the guilt and anxiety associated with hereditary disease [2,23] and the wide ranging financial and practical problems brought on by HD (not reported here) means avoiding thinking or talking about HD becomes an essential method of coping with day-to-day life. These combined factors mean that planning for future care of a relative with HD is probably beyond the carer's current capabilities or wherewithal.

This study is limited because it was based on a small sample. Future studies would benefit from accessing a larger, truly representative sample of HD carers. However, useful findings (for service provision as well as ideas for questions to be addressed in a large-scale survey) did emerge from the data.

## Conclusion

Due to the wide range of symptoms of HD, carers face many different types of psychological, emotional and practical problems, which may need a wide range of health and social services. However, patients rely heavily on informal carers rather than formal services.

The genetic implications of HD, coupled with the lack of any cure or treatment to delay the course of the disease, means that avoidance is one of the main coping strategies deployed by carers, and secrecy about the disease is manifest in HD families.

In this study avoidance was found to impair carers' abilities to plan ahead or make anticipatory arrangements for the care of future relatives who may carry the faulty HD gene. Avoidance also curtailed effective participation in any network of support that might have come from other HD carers. Avoidance and secrecy were also found to hinder support from within the immediate and wider family.

## List of abbreviations

HD Huntington's Disease

SHA Scottish Huntington's Association

## Competing interests

The author(s) declare that they have no competing interests.

## Authors' contributions

AL carried out the data collection and analysis for this study as part of her M.Sc. research. EvT supervised this research project and participated at all stages of the study. Both authors have written several drafts and approved the final manuscript.

## Pre-publication history

The pre-publication history for this paper can be accessed here:


